# The Use of Environmental DNA as Preliminary Description of Invertebrate Diversity in Three Sicilian Lakes

**DOI:** 10.3390/ani15030355

**Published:** 2025-01-26

**Authors:** Manuela Mauro, Francesco Longo, Mario Lo Valvo, Aiti Vizzini, Antonino Di Grigoli, Slobodanka Radovic, Vincenzo Arizza, Luca Vecchioni, Laura La Paglia, Vinicius Queiroz, Marialetizia Ponte, Claudio Gargano, Paolo Salvatore Francesco Ciaccio, Domenico Vicari, Mirella Vazzana

**Affiliations:** 1 Department of Biological, Chemical and Pharmaceutical Sciences and Technologies (STEBICEF), University of Palermo, Via Archirafi, 18, 90123 Palermo, Italy; manuela.mauro01@unipa.it (M.M.); francesco.long03@unipa.it (F.L.); vincenzo.arizza@unipa.it (V.A.); luca.vecchioni@unipa.it (L.V.); claudio.gargano@unipa.it (C.G.); mirella.vazzana@unipa.it (M.V.); 2 Department of Agricultural, Food and Forestry Sciences (SAAF), University of Palermo, Viale delle Scienze Ed.4, 90123 Palermo, Italy; antonino.digrigoli@unipa.it; 3 IGA Technology Services Srl., Via Linussio, 51, 33100 Udine, Italy; sradovic@igatechnology.com (S.R.); marialetizia.ponte@unipa.it (M.P.); 4 ICAR-CNR, National Research Council of Italy, Via Ugo La Malfa 153, 90146 Palermo, Italy; laura.lapaglia@icar.cnr.it; 5 Department of Physiology, Institute of Biosciences, University of São Paulo, Matthew’s House, Trav. 14, No. 101, São Paulo 05508-900, Brazil; vinicius_ufba@yahoo.com.br; 6 Independent Researcher, 92019 Sciacca, Italy; paciaccio@libero.it; 7 Experimental Zooprophylactic Institute of Sicily “A. Mirri”, 90129 Palermo, Italy; domenico.vicari@izssicilia.it

**Keywords:** biodiversity, conservation, eDNA, freshwater, metabarcoding analysis, monitoring

## Abstract

Freshwater ecosystems are among the most severely affected environments by species loss caused by climate change and intense anthropogenic pressure. To preserve biodiversity, biomonitoring plays a key role by providing reliable data on biological diversity and ecological status. Environmental DNA (eDNA) metabarcoding has emerged as a powerful and non-invasive alternative to traditional morphology-based sampling and identification methods. This study represents the first application of eDNA analysis to assess the invertebrate communities in three Sicilian Lakes: Poma, Piana degli Albanesi and Scanzano. Water samples were collected at two points in each lake and after filtration with nitrocellulose membranes, eDNA was extracted and metabarcoding analysis was performed. A total of 27 species were identified, belonging to Phyla of Annelida, Arthropoda and Rotifera. Notably, the analysis revealed the presence of alien species (*Daphnia parvula* and *Acanthocyclops americanus*), a dangerous species associated with the transmission of viral diseases (*Culex pipiens*), and potential new records for Sicily (*Stylaria lacustris*, *Platypalpus exilis*, *Pammene aurana*, *Limnephilus rhombicus*). These results provide a preliminary snapshot of invertebrate biodiversity at these sites, demonstrating how eDNA has the potential to complement, but not replace, traditional methods, contributing to the assessment of ecosystem status.

## Simple Summary

Freshwater ecosystems are among the most severely affected environments by species loss. In this context, biomonitoring plays a key role by providing reliable data on biological diversity and ecological status. Environmental DNA (eDNA) metabarcoding is a powerful and non-invasive alternative to traditional morphology-based sampling and identification methods. This study represents the first application of eDNA analysis to assess the invertebrate communities in three Sicilian Lakes: Poma, Piana degli Albanesi and Scanzano. A total of 27 species were identified, belonging to Phyla of Annelida, Arthropoda and Rotifera. Moreover, the analysis revealed the presence of alien species, dangerous species associated with the transmission of viral diseases, and potential new records for Sicily. These results provide a preliminary snapshot of invertebrate biodiversity at these sites, demonstrating how eDNA has the potential to complement, but not replace, traditional methods.

## 1. Introduction

The growing global loss of biodiversity represents one of the most serious environmental threats of our century [1,2]. An ever-increasing number of species and ecosystems are threatened by climate change and the impact of human activities [3]. Freshwater ecosystems, in particular, characterized by unique biotic and abiotic features, are amongst the most vulnerable ecosystems globally [4,5]. Lake ecosystems face a variety of environmental and anthropogenic pressures, including climate change, eutrophication, hydrological alterations, water over-extraction, habitat destruction, salinization, acidification and the introduction of invasive species, all of which pose a significant threat to their biodiversity [6,7,8,9]. The European Union, through the Water Framework Directive (WFD 2000/60/EC), seeks to prevent the deterioration of water bodies and promote the attainment of “good status” for rivers, lakes, and groundwater by establishing standards for their protection and sustainable management.

To achieve these objectives, it is essential to gather reliable data that provide detailed information on the biodiversity of these ecosystems. Monitoring biodiversity, through the identification and characterization of vertebrate and invertebrate species, could play a crucial role in understanding the effects of human activities on freshwater ecosystems [10]. Numerous studies have demonstrated that environmental DNA (eDNA) has the potential to revolutionize aquatic biodiversity monitoring, providing an efficient and non-invasive alternative to traditional morphology-based sampling and identification methods [11,12,13].

This technique typically involved four key steps: extraction, amplification, sequencing and classification of eDNA [14]. Environmental DNA refers to genetic material found in environmental samples such as sediments, soil, water, ice, and air; it is present in the form of extracellular DNA, whole cells, tissues, or, in some cases, entire organisms [15,16]. It is released into the environment from skin, mucous membranes, saliva, sperm, secretions, eggs, feces, urine, blood, roots, leaves, fruits, or pollen [17,18,19].

Once eDNA is extracted, various techniques can be used for the analysis. qPCR (Quantitative PCR) amplifies specific DNA sequences and quantifies their concentration in a sample, making it useful for detecting target species and estimating their abundance, though it is limited to known species and specific genetic markers [20,21]. ddPCR (Digital PCR) is similar to qPCR but partitions the sample into droplets, providing more precise quantification and greater sensitivity, especially for low-abundance species or rare genetic variants [22]. Lastly, metabarcoding, is a high-throughput sequencing method that identifies multiple species in a sample by targeting a specific genetic marker. Unlike qPCR and ddPCR, metabarcoding can detect a wide range of species simultaneously, providing a more comprehensive view of biodiversity without the need to target specific organisms [23]. Metabarcoding is especially valuable for environmental monitoring as it can analyze complex samples like water and soil to detect a variety of organisms, including those hard to observe with traditional methods, offering a detailed and non-invasive way to assess ecosystem health and biodiversity [17,24]. This process involves the use of general or universal polymerase chain reaction (PCR) primers on mixed DNA samples of varied origin, followed by high-throughput next-generation sequencing (NGS). This approach enables the detection of short DNA fragments used for species identification and taxonomic classification through “DNA barcoding”, a standardized DNA region [17,25,26]. The fundamental principle of DNA barcoding is based on the existence of a so-called “barcoding gap”, a specific region of the genome where variations in nucleotide sequences within a species are lower than variations observed between species [27].

The eDNA approach could offer a reliable method with significant advantages over traditional biodiversity monitoring in terms of reduced sampling invasiveness and greater time efficiency [28,29,30]. In the literature, various studies have demonstrated that this technique could be used effectively to identify vertebrate [31,32], invertebrate [33,34] and microorganism species [35]. eDNA analysis has proven particularly effective for studying freshwater invertebrates. These organisms are key in maintaining the delicate balance of freshwater eco-systems and serve as useful bioindicators of water quality [36,37]. Fernandez et al. [33], whilst testing the reliability of metabarcoding as a tool to record macroinvertebrates from a northern Iberian River (Nalón River, Asturias, Spain), observed that the molecular technique was more sensitive than traditional visual methods. However, previous studies investigating invertebrate biodiversity using eDNA have highlighted that while these techniques offer numerous advantages, they also come with certain limitations. The main advantage of eDNA lies in addressing challenges associated with morphological analysis for identifying invertebrate species. These traditional methods are often highly complex due to limiting factors such as the ability to identify only adult specimens, lengthy analysis times, and the need for advanced taxonomic expertise, which is not always readily available [38]. One of the main disadvantages is that most invertebrate Phyla possess an exoskeleton, which limits the release of eDNA into the water; this could hinder the detection of these species using molecular techniques [39]. Furthermore, the eDNA technique is subject to a number of variables that could influence the results [40]. These variables occur throughout all phases of the process, with critical points including water sampling, storage and treatment of samples, degradation processes of DNA in the environment and the use of potentially incomplete DNA barcode libraries [41,42,43,44].

Focusing on Sicily, using this technique, Hupało et al. [45] provided a preliminary overview of the freshwater diversity and community dynamics of the Fosso del Tempio River, detecting 98 macroinvertebrate species and 28 taxa potentially new to Sicily (Italy). These authors showed that diversity varied between seasons, with fewer taxa detected in winter, highlighting a dispersal barrier that had a stronger effect in autumn.

Moreover, Mauro et al. [46] used for the first time the eDNA analysis to study vertebrate biodiversity in three Sicilian lakes: Lake Poma, Piana degli Albanesi Lake, and Lake Scanzano, providing crucial information about the presence of wild species, as well as potential anthropogenic impacts.

All of these are important, considering also that the recent advancements in genetic analysis, such as the use of complete mitochondrial genomes, have further expanded our understanding of biodiversity and phylogenetic relationships. For instance, studies on the *Hirundo* genus, one of the most species-rich groups of the swallow Family, demonstrated how larger datasets of genetic material can resolve complex phylogenetic relationships and uncover new evolutionary patterns [47]. These approaches emphasize the value of high-resolution genetic tools in biodiversity research.

All studies mentioned above collectively demonstrated that eDNA metabarcoding and advanced genetic analysis provide valuable insights into biodiversity, encouraging their application in understudied regions to better understand the state and dynamics of freshwater ecosystems.

Considering these factors, the aim of this study was to evaluate, for the first time, the invertebrate community present in three significant basins in northwestern Sicily (Lake Poma, Lake Piana degli Albanesi, and Lake Scanzano) using the eDNA metabarcoding technique. The results of the analysis may provide an initial snapshot of the invertebrate species present in these sites, where existing information is currently limited. The results could offer valuable insights to support conservation strategies and sustainable management of these water resources. Furthermore, understanding and monitoring the biodiversity of these areas is essential due to their ecological significance. Lake Poma and Lake Piana degli Albanesi have been recognized by the Sicilian Region as Protection Oases and Wildlife Refuges. Lake Piana degli Albanesi is also designated as a protected area under the Habitats Directive (ITA020013 Lago di Piana degli Albanesi).

## 2. Materials and Methods

### 2.1. Lakes

**Figure 1:**
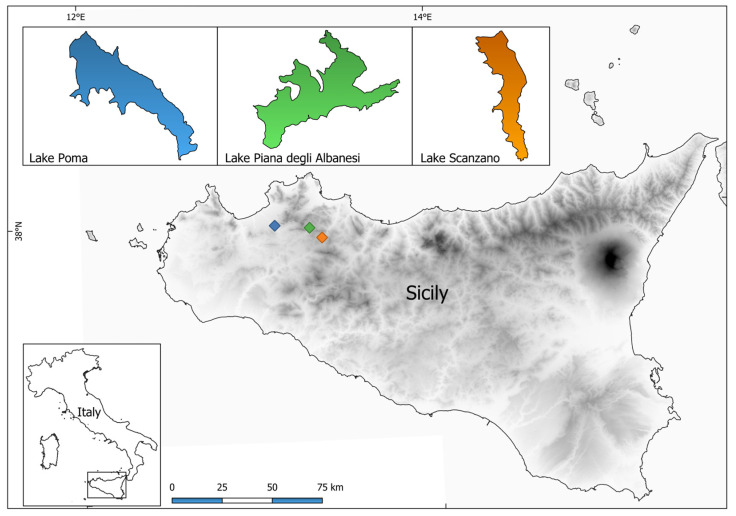
Map of the lakes in which water samples were collected.

Three different sampling sites located in northwestern Sicily were selected for this study: Lake Poma (37°59′17.45″ N–13°06′06.76″ E), Lake Piana degli Albanesi (37°58′20.54″ N–13°17′58.34″ E) and Lake Scanzano (37°55′31.84″ N–13°22′7.55″ E). All of the sites are artificial basins created through dam construction, serving as important water reservoirs for irrigation and providing potable water to nearby municipalities in the province of Palermo: Lake Poma (198 m above sea level, 269 hectares, perimeter of 11.1 km, Partinico), Lake Scanzano (518 m above sea level, 101 hectares, perimeter of 7.6 km, Piana degli Albanesi and Monreale), and Lake Piana degli Albanesi (606 m above sea level, 289 hectares, perimeter of 16.6 km, Piana degli Albanesi) (Figure 1). All three lakes are situated in agricultural areas and are fed by both meteoric and riverine inputs [31]. The different types of land use (Corine Biotopes; carta HABITAT 1:10.00000 of the Regione Siciliana) and their surface in hectares by applying a buffer of 1 km with respect to the perimeter in three Sicilian lakes are described by Mauro et al. [46].

### 2.2. eDNA Extraction

The samplings were carried out in October and November 2022. In each lake, water samples were taken at two significant points: the entry point of the inflowing stream and near the dam. Each sampling was conducted halfway down the water column, each consisting of 2 L of water. The decision to select these two sampling points per lake is based on their ability to represent the potential variability in eDNA distribution. The entry point captures external inputs, while the inflowing stream near the dam allows eDNA accumulation from the entire lake. This approach strikes a balance between feasibility and obtaining meaningful data for the first snapshot of the biodiversity.

Sterile, autoclaved glass bottles were used, each rinsed with 10% HCl and then with distilled water. After collection, the samples were stored in the dark and at low temperatures, and immediately transported to the laboratory of the Department of STEBICEF at the University of Palermo, where they were processed through filtration. Water samples were vacuum-filtered in a sterile environment (Series HC, Cheimika-HC/SLGS/F05002, Pellezzano, Italy), using nitrocellulose membranes (MF- Millipore, MCE membrane 0.22 µm, 47 mm, Merck, GSWP04700, Darmstadt, Germany). One Millipore filter was used for each 2 L sample. To prevent potential contamination during the filtration phase, funnels, tweezers, scissors and the working environment were thoroughly cleaned with 10% bleach and 96% ethanol, and decontaminated with UV light. In addition, to monitor working conditions, a control sample was prepared by filtering MilliQ water. All filters (both test and control) were cut into very thin strips approximately 1 mm wide, using tweezers and disposable scalpels [32]. eDNA extraction was carried out using DNeasy Blood & Tissue Kits (Qiagen, Hilden, Germany) following the manufacturer’s protocol, without modifications. Each DNA sample was stored at −20 °C until analysis.

### 2.3. Sequencing and Bioinformatics Analysis

IGA Technology Services Srl (Udine, Italy) carried out metabarcoding analysis on each eDNA sample. The initial step involved PCR amplification, conducted in two distinct phases. During the first phase, primers were used to amplify 142 bp of the COI region [33]. For this phase, the PCR mixture had a final volume of 25 µL and consisted of the following components: 12.5 µL of 2 × KAPA HiFi HotStart ReadyMix (Roche, Wilmington, MA, USA), 2.5 µL of the forward primer fwhF25′-GGDACWGGWTGAACWGTWTAYCCHCC-3′ (with Illumina Nextera adapter 2 µM), 2.5 µL of the reverse primer EPTDr2n 5′-CAAACAAATARDGGTATTCGDTY-3′ (with Illumina Nextera adapter 2 µM), and 7.5 µL of DNA sample. A total of 50 ng of extracted eDNA was added to this mixture and then subjected to PCR amplification under the following conditions: initial denaturation for 5 min at 95 °C, followed by 35 cycles of denaturation for 30 s at 95 °C, annealing for 1 min 30 s at 50 °C, extension for 1 min at 72 °C, and a final extension for 5 min at 72 °C. The amplified sequences thus obtained were purified using 1.6× Ampure XP beads (Beckman Coulter Life Sciences, Indianapolis, IN, USA) and eluted in 35 µL of Tris-HCl buffer pH 8.0. The second PCR was subsequently performed to incorporate index sequences, which are essential for sample demultiplexing during sequencing. The indexing PCR mixture for each sample contained 7.5 µL of the purified PCR1 amplicon, 12.5 µL of 2 × KAPA HiFi HotStart ReadyMix (Roche, Wilmington, MA, USA) and 2.5 µL of each Nextera XT index primer (Illumina, San Diego, CA, USA). This mixture was subjected to the following PCR conditions: initial denaturation for 3 min at 95 °C, 9 cycles of denaturation for 30 s at 95 °C, annealing for 30 s at 55 °C, extension for 30 s at 72 °C, and a final extension cycle for 5 min at 72 °C. After library quantification by the Qubit 1X dsDNA HS Assay Kit (Invitrogen, Thermo Fisher Scientific, Waltham, MA, USA), the indexed PCR products were equimolarly pooled and sequenced on the MiSeq platform in 2 × 300 bp mode (Illumina, San Diego, CA, USA). Base identification, demultiplexing and adapter trimming were performed using tools within MiSeq Reporter.

An internal pipeline was created to analyze the metabarcoding sequences. When the amplicon length was compatible with the sequencing read length, the 3′-ends of read pairs were overlapped using FLASH v. 1.2.11 [34] with the parameters “—max-overlap 70—min-overlap 8” to generate consensus pseudo-read. Non-overlapping read pairs were maintained as separated pairs. We retained both overlapping and non-overlapping reads. Primer sequences used to amplify the variable 12S region were removed using Cutadapt v. 2.7 [35] and with parameters “—discard-untrimmed —minimum-length 70—overlap 10—times 2—error- rate 0.15”. Low-quality bases at the 3′ ends of reads were trimmed using ERNE-FILTER v. 1.4.3 [36] and the parameters “—min-size 70”. The QIIME pipeline v. 1.9.1 [37] was then executed. The library was scanned for chimeras with the VSEARCH algorithm v. 2.14.1 [38]. The Operational Taxonomic Unit (OTU) picking process was performed in “open-reference” mode against the Eukaryote CO1 Reference Set For The Identification of DNA Metabarcodes https://github.com/terrimporter/CO1Classifier (accessed on 1 September 2024) for the RDP Classifier release v5.1.0 database [39]. This version is based on 2,216,547 COI sequences from 236,247 taxa including 185,389 species/BINs mined from GenBank and includes records deposited between 1982 and 2022 (inclusive). GenBank sequences were filtered to include only 500 bp+ sequences, free of nucleotide ambiguities, and preferably annotated with a Linnean binomial species name and/or a BOLD BIN identifier.

Taxonomy is based on the NCBI taxonomy database. Taxonomy was assigned to OTUs using the pre-defined taxonomy mapping file of the reference sequences and the RDP classifier v. 2.2 [40]. Only OTUs meeting a minimum identity threshold of 97% and a confidence threshold of 0.50 were retained for further classification. By identifying barcodes within these cleaned fragments and comparing them with reference sequences in the NCBI nucleotide database, taxonomic discrimination was achieved, ranging from the Phylum level to the Species level.

The NCBI Basic Local Alignment Search Tool (BLAST) was used for those fragments which could not be traced back to the Species level, thus enabling alignment with reference fragments from the NT database. Identification was carried out using the percentage of identity as the reference value and setting a threshold of 97%.

### 2.4. Data Analysis

After obtaining the qualitative and quantitative list of wild species present in the three Sicilian lakes, identified through eDNA, we calculated species richness (S) and biodiversity indices (H′) using the Shannon algorithm. Assemblages detected in the three lakes were then compared based on qualitative similarities using the Sorensen index, and qualitative–quantitative similarities using the Bray–Curtis and Morisita indices. To mitigate significant asymmetry in the number of fragments detected among different invertebrate taxa, we applied a base-10 logarithmic transformation to the fragment counts for calculations related to biodiversity and similarity.

## 3. Results

The metabarcoding analysis conducted on the eDNA samples collected from the three Sicilian lakes showed an average number of fragment reads as follows: 4982.0 for Lake Poma, 28,715.0 for Lake Piana degli Albanesi and 30,807.7 for Lake Scanzano. After eliminating taxonomies not belonging to invertebrate species, the average number of fragments obtained was 1005.0 for Lake Poma, 8543.0 for Lake Piana degli Albanesi and 28,246.7 for Lake Scanzano.

Identification of barcodes within these cleaned fragments and comparisons with reference sequences from the NCBI nucleotide database enabled taxonomic identification from the Phylum level up to the Species level. Results regarding Phylum, Class, Order and Family levels are summarized in Table 1, Table 2, Table 3 and Table 4. Across the three lakes, most eDNA fragments were from the Phylum Arthropoda (Table 1). Lake Poma had fewer fragments overall, all belonging to Arthropoda. In Lake Piana degli Albanesi, fragments were identified from three Phyla: Arthropoda (the majority), Rotifera, and Annelida. Lake Scanzano showed the highest number of fragments, all exclusively from Arthropoda.

Table 2 summarizes the number of cleaned fragments for which the Class level was identified. A total of six Classes were identified: three Classes in Lake Poma, five in Lake Piana degli Albanesi, and four in Lake Scanzano. Insecta was the dominant Class in both Lake Poma and Lake Scanzano. In Lake Piana degli Albanesi, a significant number of fragments attributable to the Insecta Class were also recorded; however, this lake exhibited a greater number of fragments belonging to the Eurotatoria Class, which was not identified in the other two lakes. Hexanauplia and Branchiopoda Classes were present in all lakes, with Hexanauplia showing the highest number of fragments in Lake Scanzano and Lake Piana degli Albanesi. Branchiopoda Class reached its peak in Lake Piana degli Albanesi and was minimally detected in the other two lakes. Fragments belonging to Citellata Class were identified only in Lake Piana degli Albanesi, while Arachnids appeared only in Lake Scanzano.

Table 3 presents the results of the Order-level identification. A total of 12 different Orders of invertebrates were identified: five in Lake Poma, nine in Lake Piana degli Albanesi and eight in Lake Scanzano. In both Lake Poma and Lake Scanzano, the Diptera Order had the highest number of eDNA fragments. In Lake Piana degli Albanesi, Diptera also showed high numbers, but the highest count was for the Plomia Order. Fragments from the Diplostraca, Calanoida, and Coleoptera Orders were present in all lakes, with Diplostraca peaking in Lake Piana degli Albanesi, Calanoida in Lake Scanzano, and Coleoptera in Lake Poma. Cyclopoida Order was detected only in Lake Poma and Lake Piana degli Albanesi. Other Orders were site-specific; Trichoptera appeared in Lake Piana degli Albanesi and Lake Scanzano, while Tubificida and Lepidoptera were unique to Lake Piana degli Albanesi. Lake Scanzano was the only lake where Opiliones, Hemiptera, and Neuroptera Orders were identified.

Table 4 shows the data relating to the Family identified. Across the three lakes, a total of twenty-four Families of invertebrates were detected (eight in Lake Poma; fifteen in Lake Piana degli Albanesi; fifteen in Lake Scanzano). In Lake Poma, the Chironomidae Family exhibited the highest number of detected fragments, while Syrphidae, Sididae, and Cyclopidae Families had the lowest counts. Other families detected in this lake were Diaptomidae, Staphylinidae, Culicidae, and Hybotidae. In Lake Piana degli Albanesi, the most abundant families were Trichocercidae, Diaptomidae, and Chironomidae. The least abundant families were Brachionidae, Naididae, Phoridae, Hybotidae, and Limnephilidae. Families with moderate fragment counts included Daphniidae, Cyclopidae, Haliplidae, Calliphoridae, Psychodidae, Syrphidae, and Tortricidae. In Lake Scanzano, Chironomidae had the highest count, followed by Diaptomidae, Phoridae, Simuliidae, and Micronectidae. Families with lower counts included Sididae, Phalangiidae, Daphniidae, Geotrupidae, Silphidae, Staphylinidae, Syrphidae, Hybotidae, Hemerobiidae, and Limnephilidae.

**Figure 2:**
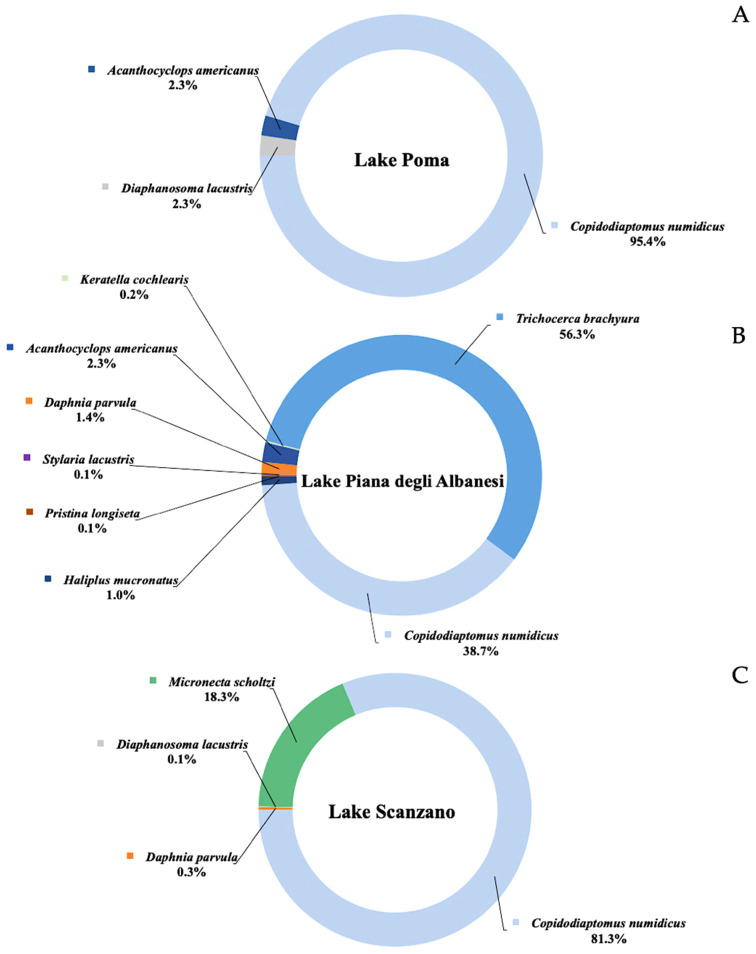
Aquatic species identified in Lake Poma (**A**), in Lake Piana degli Albanesi (**B**) and in Lake Scanzano (**C**). Results for each species are expressed as a percentage of number of fragments identified.

**Figure 3:**
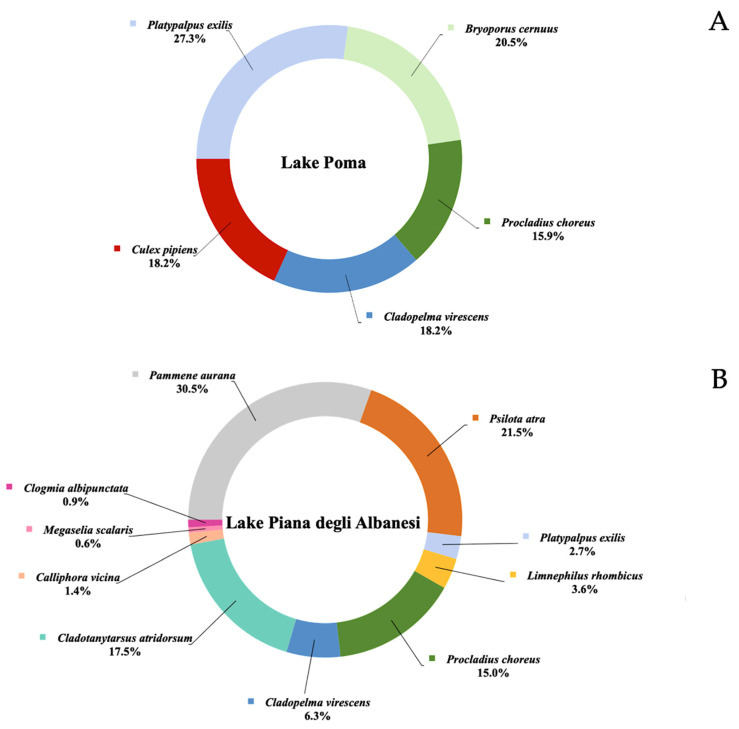

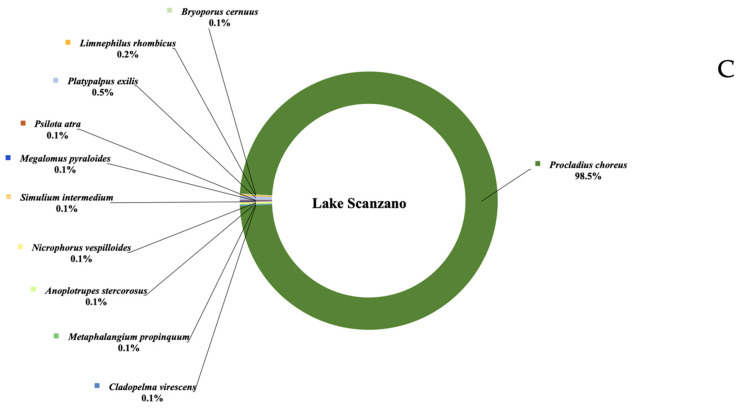
Terrestrial species identified in Lake Poma (**A**), in Lake Piana degli Albanesi (**B**) and in Lake Scanzano (**C**). Results for each species are expressed as a percentage of number of fragments identified.

Metabarcoding analysis identified eDNA fragments from 27 different species, which were divided into two categories: aquatic species (Figure 2) and terrestrial species (Figure 3). For each lake, results were presented as the percentage of average number of fragments detected for each species.

Within the category of aquatic species, three species were detected in Lake Poma, eight in Lake Piana degli Albanesi and four in Lake Scanzano. In Lake Poma, more specifically, detected species included *Copidodiaptomus numidicus* (Gurney, 1909) (95.4%), *Diaphanosoma lacustris* (Kořinek, 1981) (2.3%) and *Acanthocyclops americanus* (Marsh, 1893) (2.3%). In Lake Piana degli Albanesi, the most detected species was *Trichocerca brachyura* (Gosse, 1851) (56.3%), followed by *C. numidicus* (38.7%). The remaining species, *A. americanus*, *Daphnia parvula* (Fordyce, 1901), *Haliplus mucronatus* (Stephens, 1828), *Keratella cochlearis* (Gosse, 1851), *Stylaria lacustris* (Linnaeus, 1758) and *Pristina longiseta* (Ehrenberg, 1831), appeared in much smaller proportions.

Finally, in Lake Scanzano, as in Lake Poma, the most frequently detected species was *C. numidicus* (81.3%). Additionally, *Micronecta scholtzi* (Fieber, 1860), *Daphnia parvula* and *D. lacustris* were also identified in this lake.

The distribution of terrestrial species varied noticeably across the three lakes (Figure 3). In Lake Poma, only five species were identified, with *Platypalpus exilis* (Maigen, 1822) leading the group at 27.3%, followed by *Bryoporus cernuus* (Gravenhorst, 1806) (20.5%), and *Culex pipiens* (Linnaeus, 1758) (18.2%), both contributing significant portions, along with *Cladopelma virescens* (Meigen, 1818) (18.2%) and *Procladius choreus* (Meigen, 1804) (15.9%). Lake Piana degli Albanesi showed greater diversity, with 10 species detected. The standout was *Pammene aurana* (Fabricius, 1785), making up nearly a third of the fragments (30.5%). Other prominent species included *Psilota atra* (Loew, 1817) (21.5%), *Cladotanytarsus atridorsum* (Kieffer, 1924) (17.5%) and *P. choreus* (15.0%), while smaller percentages were contributed by species like *C. virescens*, *Limnephilus rhombicus* (Linnaeus, 1758), *P. exilis*, *Calliphora vicina* (Robineau-Desvoidy, 1830), *Clogmia albipunctata* (Williston, 1893) and *Megaselia scalaris* (Loew, 1866). Lake Scanzano was the most diverse, with 11 species identified. However, *Procladius choreus* overwhelmingly dominated, accounting for an astonishing 98.5% of the fragments. The remaining species, such as *P. exilis*, *L. rhombicus*, *C. virescens*, *Anoplotrupes stercorosus* (Scriba, 1791), *Nicrophorus vespilloides* (Herbst, 1783), *P. atra*, *B. cernuus Metaphalangium propinquum* (Lucas, 1847), *Simulium intermedium* (Roubaud, 1906) and *Megalomus pyraloides* (Rambur, 1842), appeared in much smaller proportions.

Specific richness and diversity (Table 5) were significantly lower in Lake Poma compared to Lake Piana degli Albanesi and Lake Scanzano.

**Figure 4:**
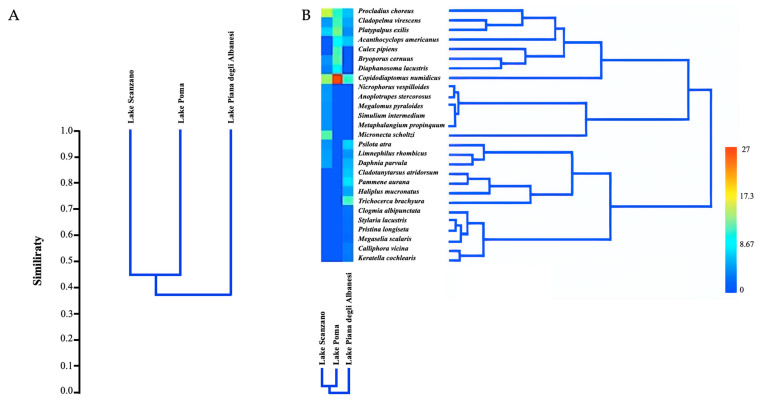
Dendrogram (**A**), dendrogram and heatmap (**B**) of the Bray–Curtis qualitative–quantitative similarity among invertebrate species in the three Sicilian lakes using the UPGMA method. The heatmap visually represents species abundances across samples, with color gradients indicating variations in abundance levels. Red indicates the highest abundance, blue represents the lowest abundance, and green indicates intermediate abundance levels.

The Bray–Curtis index revealed similarity values of less than 50% among the three lakes, indicating distinct cenoses (Figure 4A,B). Moreover, Figure 4A showed the highest qualitative and quantitative similarity in invertebrate species between Lake Scanzano and Lake Poma. A dendrogram of the Bray–Curtis qualitative–quantitative similarity among invertebrate species communities in the three Sicilian lakes was calculated by considering both species diversity (qualitative component) and the frequency of fragments detected for each species (quantitative component), using the UPGMA method. Figure 4B shows the heatmap representing species abundances across samples and variations in abundance levels. The red area indicates that the greatest abundance corresponds only to *Copidodiaptomus numidicus*.

## 4. Discussion

Due to the growing impact of human activities, it is more important than ever to continuously monitor the health of freshwater ecosystems [48]. Particularly in areas with limited information on biodiversity, data collection serves as a crucial baseline to start constant monitoring. To our knowledge, no studies have thoroughly explored the diversity of invertebrates in Lakes Poma, Piana degli Albanesi, and Scanzano. This research represents the first application of eDNA metabarcoding to analyze invertebrate diversity in these lakes. It also explores the potential of eDNA to accurately identify invertebrates and differentiate the various species present in these environments. This work builds on the overview initiated by Mauro et al. [46], who provided the first snapshot of vertebrate biodiversity in these three Sicilian lake ecosystems, classifying organisms from the Phylum level down to the Species level. A total of 27 invertebrate species were identified across the three sites, highlighting differences across the three lakes for both aquatic and terrestrial invertebrate species. This observation aligns with the findings of Mauro et al. [46], who noted differences in vertebrate species identified in the same three sites, despite high similarities in the composition of the surrounding habitats. In particular, in Lakes Poma and Piana degli Albanesi, more eDNA fragments of aquatic species were detected than terrestrial ones, whereas in Lake Scanzano, the opposite was observed. It is important to note that the number of fragments obtained could differ due to the sampling strategy, as the choice of sampling points and methods may influence the detection of specific species [49,50]. Furthermore, given the differences in species observed across the three lakes despite their habitat similarities, it could be important for future studies to consider additional physico-chemical parameters to better understand the ecological factors driving these variations.

A total of 10 aquatic species were detected across the three study sites. Among these, only *S. lacustris*, *M. scholtzi*, *H. mucronatus*, and *P. longistea* are benthic, while all the others are planktonic. The highest number of species were found in Lake Piana degli Albanesi, where fragments belonging to eight different species were identified. The only aquatic species detected in all three sites was *Copidodiaptomus numidicus*. This was the most detected aquatic species in Lakes Poma and Scanzano; furthermore, a significant number of fragments of this species were also detected in Lake Piana degli Albanesi. These results align with previous studies, confirming that *C. numidicus* is the most common calanoid species in Sicily [36,51,52,53], with its presence expanding significantly since the mid-20th century [54]. Similarly, *Diaphanosoma lacustris*, observed at multiple sites, is known to be widespread across the region (*sub D. leuchtenbergianum* [36], but see also [55,56]). The identification of *D. parvula* and *A. americanus*, two alien species of Nearctic origin, underscores the power of eDNA metabarcoding. This method proves invaluable for detecting non-native species early, helping to mitigate the serious risks they pose to biodiversity and ecosystem stability [57].

Invasive species, like *D. parvula* and *A. americanus*, for example, can disrupt local ecosystems by outcompeting native species for resources, altering habitat structures. This can lead to a decline in native biodiversity, changes in food web dynamics, and the loss of critical ecosystem functions [58,59]. The ability to detect these species early using eDNA metabarcoding is crucial for implementing timely management actions to mitigate their spread and impact, highlighting the importance of using this technique for effective ecological monitoring and conservation efforts [60,61].

However, based on pre-existing literature [36,52,53,56], some crustacean taxa, among the most widely present in the three lakes covered by this study [e.g., *Cyclops divergens* (sub *C. strenuus* in [36]), *Thermocyclops dybowsky*, *Diacyclops bicuspidatus*, *Metacyclops planus*, *Coronatella rectangula* (sub *Alona rectangula* see [36]), *Daphnia longispina*, *Daphnia ambigua*, *Ceriodaphnia quadrangula*, *Bosmina longirostris*], were not detected during the present survey, thus showing a limitation of the eDNA analysis due to temporal variability, which can affect detection, eDNA degradation caused by changes in temperatures, UV exposure or microbial activity [62,63]. Additionally, eDNA can miss rare or low-abundance species due to sampling limitations and may struggle to differentiate closely related species if the genetic markers used are not sufficiently specific [41,42,43,44].

To address these challenges, increasing the number and frequency of samples, along with employing advanced DNA extraction and amplification techniques, could help improve the detection of elusive species. The development of highly specific genetic markers and the expansion of reference databases would also be crucial to enhance taxonomic resolution and reduce identification errors. Finally, implementing spatial and temporal sampling strategies could mitigate the uneven distribution of eDNA in the environment, ensuring more reliable results [60,64,65].

Although the results obtained using eDNA can provide an initial overview of invertebrate biodiversity at these sites, it is important to consider that it is a complementary tool to traditional methods in ecosystem assessment [66]. It is clear that eDNA can provide valuable information by detecting species that are difficult to capture using conventional sampling techniques [60], but in our opinion, it is not a complete substitute for traditional methods such as visual surveys or net sampling. Traditional approaches remain crucial for obtaining direct, in situ observations of species. eDNA offers advantages such as broader spatial coverage, the ability to sample hard-to-reach areas, and the detection of species that may be missed by traditional methods, particularly in cases where organisms are present at low densities or in difficult-to-sample habitats [67,68]. However, since eDNA analysis is influenced by environmental factors, such as water movement and degradation of genetic material, its results must be interpreted with caution [69]. The combination of both approaches provides a more comprehensive and accurate assessment of biodiversity, strengthening the overall ecological monitoring strategy [70].

Other aquatic species identified in only one site include *Trichocerca brachyura*, *Haliplus mucronatus*, *Keratella cochlearis*, *Pristina longiseta*, *Micronecta scholtzi*, *Stylaria lacustris*. Their presence in Sicily has already been documented [45,55,71,72], except for *S*. *lacustris*, an aquatic oligochaete identified in Lake Piana degli Albanesi. Although this species has been previously reported in northern and southern Italy [72], it may represent a new record for Sicily. However, further investigations are needed, as no specimens were observed or collected from the site in our study.

As for terrestrial species, 17 species were detected in the three lakes, all belonging to Arthropoda. Among these, several species (*C. pipiens*, *C. albipunctata*, *S. intermedium*, *L. rhombicus*, *P. choreus*, *C. virescens*, *C. atridorsum*) have an aquatic larval stage, during which they can release eDNA into the water, particularly through molting or excretion. Due to this life cycle stage, these species could also be considered aquatic benthic organisms. However, they were classified as terrestrial species in this study because their adult phase occurs exclusively in terrestrial environments. This highlights how eDNA can detect species with complex life cycles, though it may not precisely indicate their current developmental stage or abundance. Moreover, finding DNA from terrestrial species in aquatic environments can occur for several reasons. Runoff from rain, rivers, or streams can carry soil, leaves, or other organic material into the water; the wind can also transport small particles containing DNA; terrestrial animals drinking or interacting with water sources can leave traces of their DNA in the aquatic environment [73].

Across all sites, more terrestrial species were detected compared to aquatic species. In Lake Poma, four Diptera (*Procladius choreus*, *Cladopelma virescens*, *Culex pipiens*, *Platypalpus exilis*) and one Coleoptera (*Bryoporus cernuus*) species were observed. Except for *P*. *exilis*, for which no distribution information was found in the literature for Italy, the identified species had previously been reported in other Italian freshwater systems [74,75,76]. Notably, eDNA analysis enabled the detection of *C*. *pipiens*, a mosquito species closely monitored due to its role in the maintenance and transmission of West Nile (WNV) and Usutu (USUV) viruses, Flaviviruses which significantly affect veterinary and human health [77,78]. In Lake Piana degli Albanesi, the highest number of eDNA fragments belonged to the lepidopteran *Pammene aurana*. The presence of this species at this site could represent a new taxon for Sicily and suggest an extension of the southern range limit, currently reported to be the Calabrian Region [79]. Fragments of a Trichoptera species, *Limnephilus rhombicus*, were detected. The presence of several species of Trichoptera of the genus *Limnephilus* has been reported previously in Sicily [80]; the species *L*. *rhombicus* has not yet been reported here, although its presence is known in other Italian regions, such as Lazio and Abruzzo [81]. In Lake Scanzano, the highest number of terrestrial species was identified, with 11 taxa detected. The data highlight the predominance of eDNA fragments from a single species, *P. choreus* (98.56%), yet they also emphasize the diversity of Orders and species present. This includes representatives from Coleoptera, such as *Anoplotrupes stercorosus*, *Nicrophorus vespilloides*, and *B. cernuus*, alongside Diptera species like *P. choreus*, *Cladopelma virescens*, *Simulium intermedium*, *P. atra*, and *P. exilis*. Additionally, the presence of one Neuroptera (*Megalomus pyraloides*) and one Trichoptera (*L. rhombicus*) underscores the variety of taxa that can be detected through eDNA analysis.

*Metaphalangium propinquum*, an Arachnida species belonging to the Order of Opiliones, was also found in this site. This species is widespread throughout the Mediterranean area and has also been reported in Sicily in different environments and at different altitudes [82]. Three Diptera species (*P*. *exilis*, *P*. *choreus*, and *C*. *virescens*) were identified in all three lakes. *Limnephius rhombicus* and *P*. *atra* were detected in both Lake Piana degli Albanesi and Lake Scanzano, while *B*. *cernuus* was found in Lake Scanzano and Lake Poma. Considering the short distance between the study sites, the greater homogeneity in the distribution of terrestrial species compared to aquatic ones could be attributable to the fact that some of the terrestrial species which were identified have flying adult stages, allowing for widespread terrestrial dispersal [83]. Regarding the values of specific richness and diversity, these were extremely low in Lake Poma compared to the other two lakes, in line with what was found by Mauro et al. [46] for vertebrate fauna. Despite their geographical proximity, the three reservoirs show markedly different arthropod cenoses, with one case showing significant differences in specific richness, with qualitative–quantitative similarity below 50%. These differences may depend on various factors, warranting further investigation, such as the different purposes the reservoirs are used for and the varying characteristics of the surrounding habitats that shape their composition.

## 5. Conclusions

Our results confirm the potential of eDNA to complement traditional methods in the assessment of freshwater species distribution. The results presented in this study show that the use of eDNA enabled us to identify 27 species, including alien species, dangerous species involved in disease transmission, as well as possible new records for Sicily. The use of eDNA represents an important tool to provide useful data, thus contributing to improving the understanding of biodiversity. On the other hand, the species list obtained in this study is not exhaustive since many species known to be present in these sites or observed during sampling were not detected through eDNA analysis. This could be due to various factors such as seasonality, sampling methods (littoral vs. pelagic), technical limitations (e.g., clogging of the Millipore filter often hampers proper water filtration, saturating it with DNA from species accumulating in the filter, such as microcrustaceans), the rapid degradation due to environmental factors such as temperature, UV radiation, and microbial activity.

Then, considering the current limitations of this technique, traditional taxonomic expertise remains indispensable to ensure accurate characterization of biodiversity. For this reason, the results obtained should be supported and expanded by future studies that combine eDNA analysis with conventional sampling and morphological identification techniques. The characterization of invertebrate biodiversity in these sites could be strengthened by conducting further sampling over different seasons.

## Figures and Tables

**Table 1:** Total number of cleaned fragments of Phylum identified for each lake.

Phylum	Lake Poma	Lake Piana Degli Albanesi	Lake Scanzano
Annelida	-	11.3	-
Arthropoda	1005.0	5451.3	28,246.7
Rotifera	-	3080.3	-
Total cleaned fragments	1005.0	8543.0	28,246.7

**Table 2:** Number of cleaned fragments for which the Class was identified across the three lakes.

Class	Lake Poma	Lake Piana Degli Albanesi	Lake Scanzano
Clitellata	-	11.3	-
Arachnida	-	-	17.7
Branchiopoda	10.0	120.3	36.7
Hexanauplia	220.3	2290.3	7444.0
Insecta	774.7	3040.7	20,748.3
Eurotatoria	-	3080.3	-

**Table 3:** Number of cleaned fragments for which the Order was identified.

Order	Lake Poma	Lake Piana Degli Albanesi	Lake Scanzano
Tubificida	-	11.3	-
Opiliones	-	-	17.7
Diplostraca	10.0	120.3	36.7
Calanoida	210.0	2100.7	7444.0
Cyclopoida	10.3	189.7	-
Coleoptera	75.7	55.7	58.3
Diptera	699.0	2706.7	18,791.7
Hemiptera	-	-	1854.3
Lepidoptera	-	250.0	-
Neuroptera	-	-	17.3
Trichoptera	-	28.3	26.7
Ploima	-	3080.3	-

**Table 4:** Number of cleaned fragments for which the Family was identified.

Family	Lake Poma	Lake Piana Degli Albanesi	Lake Scanzano
Naididae	-	11.3	-
Phalangiidae	-	-	17.7
Daphniidae	-	120.3	27.7
Sididae	10.0	-	9.0
Cyclopidae	10.3	189.7	-
Diaptomidae	210.0	2100.7	7444
Geotrupidae	-	-	20.7
Silphidae	-	-	20.0
Staphylinidae	75.7	-	17.7
Haliplidae	-	55.7	-
Calliphoridae	-	120.0	-
Chironomidae	574.0	2298.3	16,913.7
Culicidae	66.7	-	-
Phoridae	-	36.3	758.3
Psychodidae	-	61.7	-
Simuliidae	-	-	1014.0
Syrphidae	8.3	169.0	18.0
Hybotidae	50.0	21.3	87.7
Micronectidae	-	-	1854.3
Tortricidae	-	250.0	-
Hemerobiidae	-	-	17.3
Limnephilidae	-	28.3	26.6
Brachionidae	-	10.0	-
Trichocercidae	-	3070.3	-

**Table 5:** Specific richness and diversity in three Sicilia Lakes.

	Lake Poma	Lake Piana Degli Albanesi	Lake Scanzano
Taxa_S	8	18	15
Shannon_H	2.039	2.877	2.652

## Data Availability

Non-public data for privacy; contact the authors.
